# Overestimation of the probability of death on peritoneal dialysis by the Kaplan-Meier method: advantages of a competing risks approach

**DOI:** 10.1186/1471-2369-13-31

**Published:** 2012-05-30

**Authors:** Jean-Baptiste Beuscart, Dominique Pagniez, Eric Boulanger, Celia Lessore de Sainte Foy, Julia Salleron, Luc Frimat, Alain Duhamel

**Affiliations:** 1 CERIM, EA 2694, Faculty of Medicine, Research Department, 1 Place Verdun, F-59045, Lille, CEDEX, France; 2 University of Lille, Lille, France; 3 Department of Biostatistics, EA2694, UDSL, Lille, France; 4 Nephrology Department, CHU, Lille, France; 5 Nephrology Department, EA 4003, INSERM CIC-EC CIE6, Nancy University, Nancy, France

## Abstract

**Background:**

In survival analysis, patients on peritoneal dialysis are confronted with three different outcomes: transfer to hemodialysis, renal transplantation, or death. The Kaplan-Meier method takes into account one event only, so whether it adequately considers these different risks is questionable. The more recent competing risks method has been shown to be more appropriate in analyzing such situations.

**Methods:**

We compared the estimations obtained by the Kaplan-Meier method and the competing risks method (namely the Kalbfleisch and Prentice approach), in 383 consecutive incident peritoneal dialysis patients. By means of simulations, we then compared the Kaplan-Meier estimations obtained in two virtual centers where patients had exactly the same probability of death. The only difference between these two virtual centers was whether renal transplantation was available or not.

**Results:**

At five years, 107 (27.9%) patients had died, 109 (28.4%) had been transferred to hemodialysis, 91 (23.8%) had been transplanted, and 37 (9.7%) were still alive on peritoneal dialysis; before five years, 39 (10.2%) patients were censored alive on peritoneal dialysis. The five-year probabilities estimated by the Kaplan-Meier and the competing risks methods were respectively: death: 50% *versus* 30%; transfer to hemodialysis: 59% *versus* 32%; renal transplantation: 39% *versus* 26%; event-free survival: 12% *versus* 12%. The sum of the Kaplan-Meier estimations exceeded 100%, implying that patients could experience more than one event, death and transplantation for example, which is impossible. In the simulations, the probability of death estimated by the Kaplan-Meier method increased as the probability of renal transplantation increased, although the probability of death actually remained constant.

**Conclusion:**

The competing risks method appears more appropriate than the Kaplan-Meier method for estimating the probability of events in peritoneal dialysis in the context of univariable survival analysis.

## Background

Patients with stage 5 chronic kidney disease (CKD) can be treated by peritoneal dialysis (PD), hemodialysis (HD) or renal transplantation. The efficacy of PD is frequently assessed from the patient survival, estimated by the Kaplan-Meier method and compared to patient survival on HD using the Cox proportional-hazards model [1,2]. In these survival analyses, patients on PD may encounter three outcomes: transfer to HD, renal transplantation, or death. The Kaplan-Meier method can only take into account one of these events. The validity of the estimations made using this approach for patients on PD is therefore questionable.

A competing risk is an event which either hinders the observation of the event of interest, or modifies its probability of occurrence [3,4]. In the survival analysis of PD patients, renal transplantation or transfer to HD may hinder the observation of death [1,2,5]. Transplantation or transfer to HD should therefore be considered as competing risks, which may influence the calculations and therefore the results of the survival analysis [6].

Analysis of time-to-event data when competing risks are present requires specific methods because standard approaches can lead to estimation and interpretation errors [3,7,8]. For instance, in other fields of medicine such as cancer research and cardiology, or even in renal transplantation, the Kaplan-Meier method has been shown to overestimate the probability of death in comparison with the more specific competing risks method [8-11].

The purpose of the present study was to assess the validity of the survival estimations in PD obtained by the Kaplan-Meier method compared to the competing risks method developed by Kalbfleisch and Prentice in a cohort of 383 PD patients. The Kaplan-Meier method is often considered to estimate the survival that would be observed in the absence of transfer to HD or transplantation. We used simulations to investigate this assumption, and also tested the competing risks approach under the same conditions.

## Methods

### Patients

This study was performed at the Lille University Medical Center Nephrology Department (France). All consecutive incident patients starting PD treatment between January 1, 1992, and July 1, 2007 were included in the study. The cut-off date was January 1, 2008. Data on age, gender, diabetic status and primary renal diagnoses were collected at baseline. The primary renal diagnosis was classified according to the French renal epidemiology and information network [12]. Follow-up ended in the event of death, transfer to HD or renal transplantation, transfer to another center, recovery of renal function, or at termination of the study. The study was approved by the Ethics Committee for Medical Research of the university hospital of Lille (Correspondant Informatique et Liberté, number 701012-GD).

### Statistical methods

The current paper only focuses on univariate methods for survival analysis. Two approaches can be used in the survival analysis of PD patients. In the intention-to-treat approach, death is taken into account if it occurs during PD or after transfer to HD [1,2]. In this approach, renal transplantation is a competing risk. In the as-treated approach, death is only taken into account if it occurs during PD. In this approach, both transplantation and transfer to HD are competing risks [5]. The analyses of real data were performed according to an as-treated approach, in order to illustrate the three different events that could occur in PD patients: death, transfer to HD, or renal transplantation.

#### Estimation of event-free survival and cumulative incidences

Event-free survival is the probability of being free from any event, which corresponds here to the probability of staying alive on PD. All events are taken into account in this survival estimation, so that there is no competition between events. It can be assessed by the Kaplan-Meier method [4,7]. The events analyzed in the study were death, transfer to HD, and renal transplantation. Alive at cut-off date, transfer to another center, and recovery of renal function were censored.

**Figure 1:**
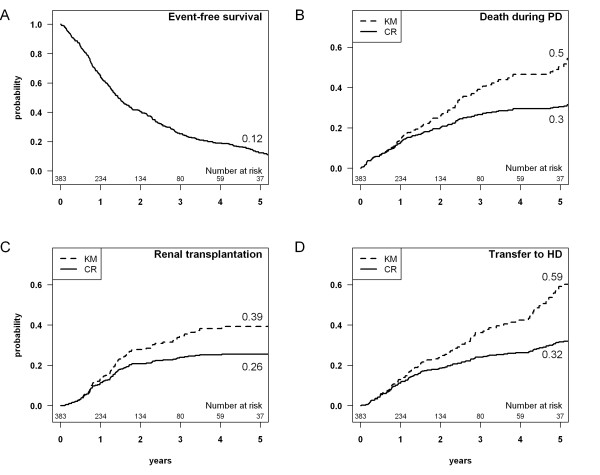
**A: Event-free survival, which corresponds to the probability of staying alive on peritoneal dialysis (PD). B-D:** Cumulative incidence estimations obtained by the Kaplan-Meier (KM) and the competing risks (CR) methods for: death during PD; renal transplantation; transfer to hemodialysis (HD).

**Figure 2:**
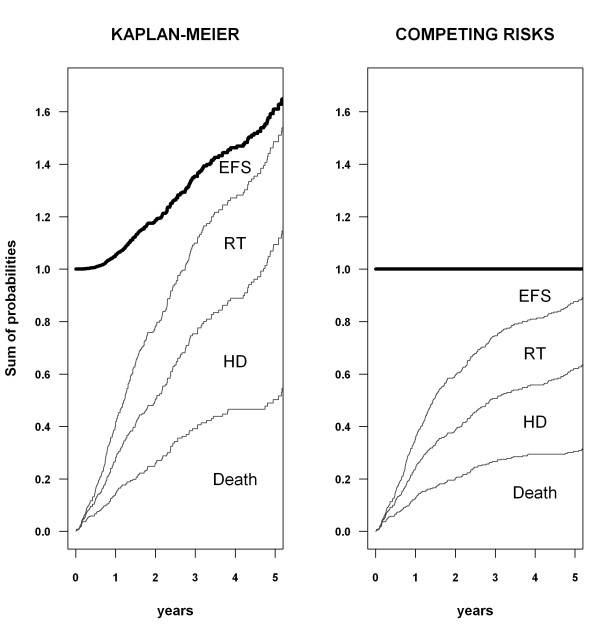
**Sum of probabilities estimated by the Kaplan-Meier and competing risks methods.** Event-free survival (EFS) and the cumulative incidence curves for death, transfer to hemodialysis (HD), and renal transplantation (RT) are stacked. The upper line (in bold) represents the sum of probabilities of the different events.

**Figure 3:**
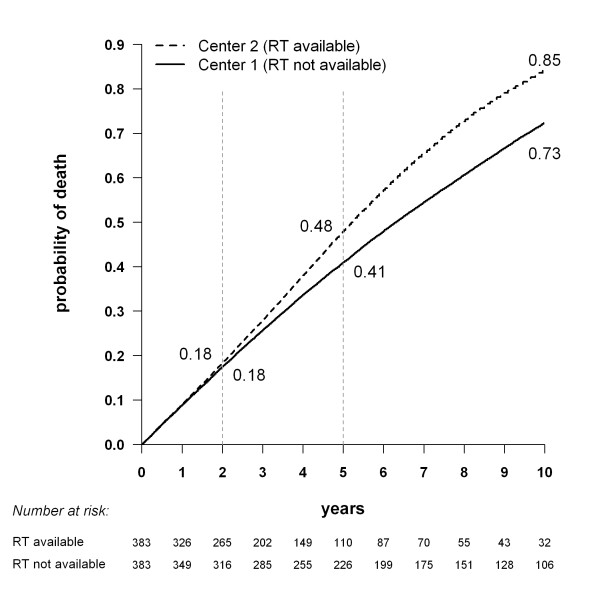
**Cumulative incidence of death for two simulated dialysis populations with exactly the same probability of death, obtained by the Kaplan-Meier method.** The only difference between the two virtual centers is whether renal transplantation (RT) is available (center 2) or not (center 1).

**Figure 4:**
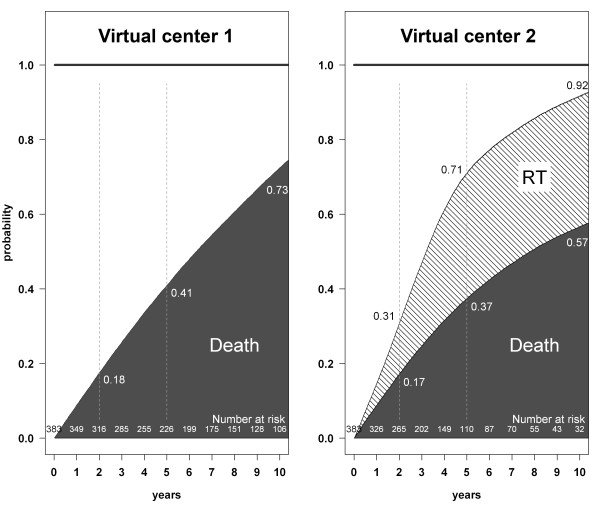
**Cumulative incidence of death and renal transplantation for two simulated dialysis populations with exactly the same probability of death, obtained by the competing risks method.** The only difference between the two virtual centers is whether renal transplantation (RT) is available (center 2) or not (center 1). Dashed vertical lines show the estimates at 2, 5, and 10 years for each center.

The cumulative incidence function of cause *k* is defined by the probability of failing from cause *k* before time *t*[3,7]. The cumulative incidence function was estimated by both the Kaplan-Meier and the competing risks methods for the following causes: death during PD, transfer to HD, renal transplantation. With the Kaplan-Meier method, only the event of interest was taken into account and all other events were censored at the time of the event. For example, to estimate the cumulative incidence of death during PD, the following events were censored: transfer to HD and renal transplantation. With the competing risks method, death during PD, transfer to HD, and renal transplantation were considered as competing risks. The cumulative incidence function was estimated for each of these outcomes using the approach of Kalbfleisch and Prentice, which takes into account all events in the calculations made through event-free survival [4]. As recommended, the duration shown on the curves (Figures 1, 2, 3, and 4) was stopped if less than 10% of the patients were still under follow-up [13]. We conducted a sensitivity analysis by separating the cohort into two subgroups according to the date of inclusion: early inclusion between 1992 and 1999, and late inclusion between 2000 and 2007.

#### Sum of probabilities

Death, transfer to HD, or renal transplantation were the only three events that could occur in a given patient. In their absence, patients were still alive on PD. By definition, these four states are mutually exclusive: a patient cannot simultaneously die and be transferred to HD, or be alive on PD and have a renal transplant. Therefore, at all time points, the sum of these probabilities must be equal to one.

### Simulations with or without the availability of renal transplantation

The aim was to compare the survival estimates obtained for two identical cohorts, one with the presence and the other with the absence of competing risks. Such real data are not available for dialysis, because randomized controlled trials comparing dialysis alone to dialysis plus transplantation have never been carried out for ethical reasons. We therefore simulated two different situations in two virtual centers. To simulate an intention-to-treat analysis, which is the preferred approach in the survival analysis of patients on PD, two events only were taken into account: death or renal transplantation. The simulation was based on specific characteristics (age and the presence or not of diabetes) of the 383 patients in our cohort.

In virtual center 1, renal transplantation was not available. We used a Cox-Gompertz model to simulate the survival time for each patient [14]. The parameters of the model were calculated to closely simulate data from Japan, where the renal transplantation rate is low [15]. We set a similar hazard ratio (*HR*) for death to that obtained for our cohort using a Cox model: *HR* = 2 for diabetes, *HR* = 3 for age between 60 and 75 years, *HR* = 9 for age over 75 years.

In virtual center 2, patients had the same survival times as in center 1, the difference being that renal transplantation was available. It has been reported that patients on the waiting list have a higher survival time than other patients [16], while age and the presence of diabetes have been shown to influence access to renal transplantation [17,18]. Therefore, for each patient *i*, the probability *pi* of being placed on the waiting list decreased with older age, the presence of diabetes, and lower survival time. Registration status (on the waiting list or not) was then generated for each patient *i* using a Bernoulli distribution for parameter *pi*. For patients on the waiting list, waiting times were simulated with a Gompertz model. All parameters were set to closely simulate registration on the waiting list and waiting times in France [19]. If the waiting time was shorter than the survival time, renal transplantation was observed. If the waiting time was longer than the survival time, or if the patient was not on the waiting list, death was observed with the same survival time as in that in center 1.

We compared the Kaplan-Meier estimations made for the two virtual centers with the Log rank test. To avoid a biased conclusion reached on the basis of a single simulation, the latter was repeated 100 times and the results were averaged. Six different simulations (repeated 100 times) were carried out to obtain six different proportions of patients on the waiting list in center 2: 5, 10, 20, 30, 40, and 50% of the population on dialysis. Lastly, we used the Kalbfleisch and Prentice method to estimate the cumulative incidences of death and renal transplantation in the two virtual centers.

#### Software

All statistical calculations were carried out using R software (R Development Core Team [2009], R: A language and environment for statistical computing) with the “Survival” and “Cmprsk” packages.

## Results

### Patients and observed outcomes

Three hundred and eighty-three consecutive incident patients were included in the study. Patient characteristics were presented in Table 1. At five years, 107 patients (27.9%) had died during PD treatment, 109 (28.4%) had been transferred to HD, 91 (23.8%) had undergone renal transplantation, and 37 (9.7%) were still on PD. The other 39 (10.2%) patients had a shorter duration of follow-up, and their data had been censored when they were still alive on PD (33 patients), upon transfer to another center (three patients), or after recovery of renal function (three patients). The median follow-up period was 15.7 months (interquartile range: 8.6–24.7).

**Table 1:** Baseline characteristics of the 383 patients included in the study

**Patient characteristics**	**Patients (n = 383)**
Age, yr [mean (SD)]	56.5 (18.1)
Women	160 (41.8%)
Diabetes	100 (26.1%)
Primary renal disease	
Glomerulonephritis	121 (31.6%)
Diabetes	54 (14.1%)
Vascular	44 (11.5%)
Pyelonephritis	34 (8.9%)
High blood pressure	16 (4.2%)
Polycystic kidney disease	15 (3.9%)
Other	51 (13.3%)
Unknown	48 (12.5%)

SD: standard deviation.

### Event-free survival and cumulative incidence estimations

Death was taken into account during PD only, in terms of as-treated analysis. Event-free survival, which corresponds to the probability of staying alive on PD, was estimated at 12% at five years, as shown in Figure 1A. The probability of remaining alive on PD decreased rapidly due to the high rate of events, evenly distributed between death, transfer to HD, and renal transplantation.

Cumulative incidence curves for each event estimated by the Kaplan-Meier and Kalbfleisch and Prentice methods are shown in Figure 1B–D. With the Kaplan-Meier method, the cumulative incidence estimations for death during PD, transfer to HD, and renal transplantation were systematically higher than the observed proportion of events: 50% vs 27.9%, 59% vs 28.4%, and 39% vs 23.8%, respectively. Conversely, with the competing risks method, at five years, the cumulative incidence estimations for death during PD, transfer to HD and renal transplantation amounted to 30%, 32% and 26% respectively. This finding was in accordance with the observed proportion of events, taking into account censored observations.

With the Kaplan-Meier method, the 109 patients transferred to HD and the 91 renal transplantations were censored to estimate the probability of death during PD. These 200 patients (51.6%) were considered to have the same risk of dying during PD as those patients still on PD. This led to a difference in the estimation of the cumulative incidence of death between the Kaplan-Meier method and the competing risks method, which amounted to 20% at five years. The sensitivity analysis showed that the results were similar for death and transfer to HD between the early- and late-inclusion patients (data not shown).

The sums of the reported probabilities at time *t*, estimated using both methods, are shown in Figure 2. With the Kaplan-Meier method, this sum rapidly exceeded one. At five years, it amounted to 160%: 12% of patients would be still alive on PD, 50% would have died, 59% would have been transferred to HD, and 39% would have been transplanted. This meant an expected number of events per patient that was greater than one at five years, which is not possible. In contrast, the sum of probabilities using the competing risks method amounted to one at all times.

### Simulations

Simulations were used to compare the survival estimations obtained for two identical cohorts in different competing risks settings. We simulated two virtual centers where patients had exactly the same characteristics and the same probability of death. The only difference between these two virtual centers was whether renal transplantation was available (center 2) or not (center 1). Death was taken into account on both PD and HD, according to an intention-to-treat analysis. One hundred simulations were carried out, and the results were averaged. In virtual center 1, where renal transplantation was not available, the median survival time was 6.3 ± 0.3 years. In virtual center 2, the mean number of patients on the waiting list was 145 (38%) ± 7 patients. The median waiting time before transplantation was estimated at 2.5 ± 0.2 years.

The cumulative incidence of death estimated by the Kaplan-Meier method is shown in Figure 3. The probability of death was exactly the same in the two virtual centers but the estimated cumulative incidence of death was significantly higher in virtual center 2, where transplantation was available (*P* < 0.001 for each simulation). In the latter center, 131 patients underwent renal transplantation during the first 10 years. These observations were censored at the time of transplantation. In virtual center 1, the 131 corresponding patients were long-term survivors, as 105 of them remained on dialysis for more than five years, and 60 of them for more than 10 years.

The influence of censoring renal transplantation in the Kaplan-Meier estimations increased with the proportion of patients on the waiting list. The gap between the two cumulative incidence curves increased from 1.5% to 18% at ten years, when the proportion of patients on the waiting list had increased from 5% to 50% (data not shown).

With the competing risks method, the cumulative incidence of death was systematically lower in virtual center 2 than in virtual center 1, as shown in Figure 4. After transplantation, patients in virtual center 2 were no longer considered at risk of death during dialysis. If the curves had been continued until the last death event, the cumulative incidence of death would have reached 100% in virtual center 1 and 65% in virtual center 2, the 35% difference between the two centers corresponding to the transplanted patients.

## Discussion

In our study, the Kaplan-Meier method overestimated the probability of each event, i.e. death, transfer to HD, or renal transplantation during PD. This approach takes only one event into account, the other events being censored [3,4]. When the event investigated was death, patients censored because of transfer to HD or renal transplantation were considered to be withdrawn alive on PD, which led to an overestimation of the probability of death during PD. When the event studied was transfer to HD or renal transplantation, patients who died were censored and considered to be withdrawn alive on PD. The sum of probabilities thus exceeded one, implying that more than one event could occur in a given patient. This is not possible in real terms, as for example a patient cannot first die during PD then later have a renal transplantation.

The competing risks method provided accurate estimations of event probabilities when applied to our study cohort. In particular, the sum of the estimated probabilities amounted to one at all times. Our results showed that in the PD setting, crude cumulative incidences of each event could be estimated by means of the Kalbfleisch and Prentice method. Using this approach, patients who experienced an event were no longer at risk of death during PD. Consequently, cumulative incidence curves should not be interpreted alone [4,7]. For instance, the interpretation of the cumulative incidence of death must take into account the cumulative incidence of renal transplantation and transfer to HD.

The Kaplan-Meier method is frequently considered to estimate the virtual survival rate in PD, i.e. that which would be observed in the absence of any competing risk. However, the results of our simulations were not consistent with this assumption. The probability of death estimated when renal transplantation was censored (center 2) was systematically higher than that estimated when renal transplantation was not available (center 1). This was due to the independence assumption underlying the censoring process, which assumes that individuals censored at time t have the same probability of developing the event of interest beyond time t as those who remain in follow-up [4,7,20]. Patients excluded because of renal transplantation were thus assumed to have the same risk of death as patients still in follow-up. This assumption does not hold true for dialysis: patients on the waiting list are younger, healthier, and have a better chance of survival than other patients [16-18]. These results revealed a second problem when dealing with the probability of death on PD, namely that the case-mix of patients remaining in the study changes when the patients who are relatively healthy undergo renal transplantation, leaving those with a worse prognosis on dialysis.

The use of the competing risks method is recommended in clinical settings where dependent events are present [3,4,7]. However, the method of Kalbfleisch and Prentice is also unable to estimate the virtual survival rate in PD. In our simulations, the probability of death estimated by this method was indeed lower when renal transplantation was available (center 2) than when it was not (center 1). The estimations of cumulative incidence of death made by the Kalbfleisch and Prentice method in virtual center 2 were correct, but they cannot be interpreted alone. In fact, the lower incidence of death observed in Figure 4 in center 2 was the consequence of a high incidence of renal transplantation in this virtual center. Transplantation has been shown to offer a survival advantage compared to dialysis, and the lower probability of death on dialysis might be considered a successful outcome [16,21]. Taken as a whole, these results suggest that the competing risks method should become the method of choice in PD survival analysis. The use of competing risks methods may also be considered in nephrology in general, as the problem of competing risks occurs frequently in many other groups of patients. For example, the competing risk of renal transplantation is also a factor to be taken into consideration when analyzing survival of HD patients, or the competing risk of dialysis initiation when analyzing survival of patients with chronic kidney disease, or the competing risk of death with a functioning transplant when analyzing the allograft survival in transplanted patients.

The efficacy of PD is frequently assessed on the basis of patient survival and technical survival, estimated by the Kaplan-Meier method. The transplantation rate varies greatly between countries or centers [17,22]. As shown in our study, centers with a high transplantation rate may be penalized if their results for dialysis are represented by these estimations. To compare PD centers, it would be useful to estimate the survival observed in the absence of transfer to HD and renal transplantation. To estimate this survival in a dependent, competing risks setting such as PD, fairly strong assumptions have to be made and more sophisticated methods are required [23,24]. The Kaplan-Meier method cannot achieve this, and should only be used to estimate event-free survival [4,7].

We applied the Kaplan-Meier and the competing risks methods to a single-center cohort of 383 consecutive incident patients, as recommended in the literature [25,26]. Since nearly all events were observed over a 16-year period of follow-up, with only 10.2% observations being censored before five years, these results could be considered reliable. The observed proportion of deaths, transfer to HD, and transplantations was similar to that reported for other European cohorts of incident PD patients, making our cohort a valid basis for comparison [27-29]. We used as-treated analysis to process our observed data, and intention-to-treat analysis for our simulations. In the literature, both approaches may be used depending on the aim of the study [1,2,5].

Randomized controlled trials comparing dialysis alone to dialysis plus transplantation are viewed as unethical, and so have never been carried out. Data on patients for whom access to transplantation would constitute the only difference are therefore not available. Our response to this situation was to make use of simulations. We used a Gompertz model, which appears to be more appropriate than the exponential or Weibull models [14], and allowed us to make simulations based on real data. However, a simulation cannot provide real observed data, an aspect which should be kept in mind at the time of interpretation. A case in point is that the exact form of the dependence function between survival and registration for transplantation is unknown. Expert elicitation would be useful in this regard, as for instance in environmental health where the knowledge-base is limited by incomplete data [30].

As the aim of our study was to compare the two methods in general, and in particular to analyze the influence of competing risks on Kaplan-Meier estimates, the question of bivariate and multivariate analysis in a competing risks setting was not addressed here.

## Conclusion

Our study has shown that the Kaplan-Meier method overestimated the probability of death in the competing risks setting of PD. The use of the competing risks method is recommended in survival analysis when several dependent events are possible. This approach is used in other fields of medicine, such as hematology [31,32]. It has also occasionally been used in nephrology, renal transplantation, and in studying peritonitis-freesurvival in PD [6,11,33]. We suggest that the competing risks method should be adopted as the preferred approach in PD univariable survival analysis.

## Competing interests

The authors declare that there are no competing interests.

## Authors’ contributions

JBB and AD designed the study. DP, EB, and CL acquired the data. JBB and DP obtained funding. JBB, JS, and AD performed the statistical analysis. JBB, DP, and AD drafted the manuscript. EB, CL, and LF revised the manuscript for important intellectual content. JBB, DP, EB, CL, JS, LF, and AD gave their final approval regarding submission for publication. All authors read and approved the final manuscript.

## Pre-publication history

The pre-publication history for this paper can be accessed here:

http://www.biomedcentral.com/1471-2369/13/31/prepub
